# Identification and Quantification of Fatty Acids in* T. viridissima*,* C. biguttulus*, and* C. brunneus* by GC-MS

**DOI:** 10.1155/2018/3679247

**Published:** 2018-02-28

**Authors:** Alexander M. Wathne, Hanne Devle, Carl Fredrik Naess-Andresen, Dag Ekeberg

**Affiliations:** Faculty of Chemistry, Biotechnology and Food Science, Norwegian University of Life Sciences, P.O. Box 5003, 1432 Ås, Norway

## Abstract

Fatty acid (FA) profiles of the species* Tettigonia viridissima*,* Chorthippus biguttulus*, and* Chorthippus brunneus* were determined and quantitated. Extracted lipids were derivatized into FA methyl esters (FAMEs) prior to analysis by GC-MS. A total of 37 different FAs were identified in* T. viridissima*, yielding a total FA content of 10.4 g/100 g of dry matter. The contents of saturated FAs, monounsaturated FAs, and polyunsaturated FAs were 31.1, 35.9, and 33.0%, respectively. Lipids from* T. viridissima* were also fractioned into neutral lipids, free fatty acids, and polar lipids by offline solid phase extraction. For* C. brunneus *and* C. biguttulus*, 33 FAs were identified, yielding a total FA content of 6.14 g/100 g of dry matter. SFAs, MUFAs, and PUFAs, respectively, constituted 32.7, 25.1, and 42.1% of the total FA content. The contents of MUFAs, PUFAs,* n*-3 FAs, and* n*-6 FAs of each species, and the* n*-6/*n*-3 ratio, were subsequently discussed.

## 1. Introduction

As the world's population surges towards a total of 9 billion people by the middle of the 21st century, an increased global demand for food will inevitably follow. Additionally, a higher consumption of beef, fish, and poultry will be facilitated by the higher purchasing power of the emerging middle class in developing countries, resulting in an increased pressure on the food supply system [1]. An estimated 800 million people still experience hunger around the globe, chronically or transitionally [2]. To face the daunting task of feeding the growing population and those currently lacking basic nutrition, novel and more efficient foods will have to be studied and consequently utilized for human consumption on an industrial scale. Insects play an important role in human nutrition outside Europe in areas such Asia, Africa, and South-America, functioning as a nutritionally viable alternative to meats and fish [3]. Moreover, insects also provide important micronutrients such as calcium, iron, and zinc [4]. Approximately 13% of the insects consumed globally belong to the order Orthoptera, which includes grasshoppers, crickets, and locusts [4].

Essential fatty acids (EFAs) are defined as FAs essential to growth and development [5]. These EFAs must be supplied through the diet, as they are not synthesized by the human body. Linoleic acid (LA) and *α*-linolenic acid (ALA), an* n*-6 and* n*-3 FA, respectively, have been identified as the two EFAs required to be included in the diet [6]. Both are precursors to the* n*-6 FA arachidonic acid (AA), but only ALA acts as a precursor to the important* n*-3 FAs eicosapentaenoic acid (EPA) and docosahexaenoic acid (DHA), both of which are produced through biosynthesis by elongation and desaturation [7]. DHA and EPA have been associated with proper retinal and immune function and are present in significant quantities in both the brain and the nervous system [8–10]. Deficiencies of EPA and DHA have been linked to several chronic diseases and disorders [11, 12]. Furthermore, low levels of dietary ALA have been associated with overall deficiencies of both DHA and EPA [13].

The intake ratio of* n*-6 to* n*-3 FAs through diet has also been reported to be of significance in overall health [14–17], with ratios ranging from 1/1 to 2/1 being highlighted as optimal. Western societies in particular have a high intake of* n*-6 FAs compared to* n*-3 FAs [18]. A high intake of* n*-6 FAs results in increased levels of proinflammatory, prothrombotic, and proaggregatory eicosanoid secondary metabolites at the expense of anti-inflammatory and hypolipidemic eicosanoids derived from* n*-3 FAs [5, 19]. However, it should be noted that the FAO does not give any specific recommendations for this ratio and that the importance of this ratio is debated [6].

In studies evaluating insects as a part of human diet one should consider the crucial role of FAs, and in particular the influence of EFAs on human health. The FA composition will vary with individual species, their development phase, diet, and environmental factors [20]. However, few efforts have been made to explore the composition of fats in grasshoppers [20]. The study of Paul et al. [21] elucidated the FA contents of three Orthopterans,* Acheta domesticus* (Gryllidae family),* Conocephalus discolor *(Tettigoniidae family), and* Chorthippus parallelus* (Acrididae family), by utilizing GC-FID. Their results revealed high concentrations of LA and ALA, as well as oleic acid. Yang et al. [22] studied the FA profiles of three Orthopterans (Gryllidae and Acrididae family) native to Thailand, also revealing significant concentrations of these FAs. Additionally, in their study the spur-throated grasshopper,* Chondracris roseapbrunner* (Acrididae family), displayed a favourable* n*-6/*n*-3 ratio of 0.3/1.

Fractioning of FAs from insects by use of offline solid phase extraction (SPE) appears to be lacking in the literature. Grapes et al. [23] fractionated the lipids extracted from organs in adult females belonging to* Acheta domesticus* into three classes: neutral lipids (NLs), polar lipids (PLs), and free fatty acids (FFAs). In their study, diacylglycerides and triacylglycerides from the NL fraction were found to be present in major concentrations.

The objectives of this study were to identify and quantitate FAs present in the grasshopper species* Chorthippus brunneus *and* Chorthippus biguttulus* (both from the Acrididae family,* Chorthippus* genus), as well as the bush cricket* Tettigonia viridissima* (Tettigoniidae family).* C. brunneus *and* C. biguttulus* were treated as a single species* (Chorthippus *sp.), as they are morphologically undistinguishable [24, 25]. All three species belong to the order Orthoptera and are commonly found in southern Scandinavia, continental Europe, temperate Asia, and parts of northern Africa. To evaluate the potential health benefits by incorporating these insects into the diet, saturated fatty acids (SFAs), monounsaturated fatty acids (MUFAs), polyunsaturated fatty acids (PUFAs),* n*-3, and* n*-6 FAs were the focus of the study, as well as the* n*-6/*n*-3 ratio.

To the knowledge of the authors, the work presented in this article is the first study conducted to elucidate and quantitate the complete FA profiles of the species* T. viridissima*,* C. biguttulus*, and* C. brunneus*, as well as the first use of SPE for extracted FAs from complete, homogenized specimens.

## 2. Materials and Methods

### 2.1. Chemicals and Standards

The chloroform used for lipid extraction from the sample tissues, and internal standard (IS) stock solutions, was supplied by VWR Chemicals and of Chromanorm quality (France). Methanol, used in conjunction with chloroform for the extraction procedure and to make the sodium methoxide solution, was supplied by Sigma-Aldrich and of Chromasolv quality (France). The 10% (1.3 M) boron-trifluoride-methanol solution used for transesterification of the lipids to FAMEs was supplied by Sigma-Aldrich (Switzerland). Heptane ≥ 99% n-heptane basis (GC) was supplied by Sigma-Aldrich (Israel). Acetic acid and diethyl ether were used in combination as a mobile phase for the elution of FFAs by offline SPE. The acetic acid 96% puriss. p.a. was supplied by Riedel-de Haën (Germany) and the diethyl ether puriss. p.a. ≥ 99,8% was supplied by Sigma-Aldrich (Poland).

A total of five internal standards were chosen for quantitation of the FAMEs. They were all supplied by Larodan AB (Malmö, Sweden): undecanoic acid (C11:0 FFA), triundecanoin (C11:0 TG), nonadecanoic acid (C19:0 FFA), trinonadecanoin (C19:0 TG), and 1,2-dinonadecanoyl-sn-glycero-3-phosphatidylcholine (C19:0 PL). The IS stock solutions were all prepared by dissolving 10 mg of standard with chloroform to a final concentration of 1.0 mg/mL, with the exception of C11:0 TG, which was prepared by dissolving 1 mg of standard with chloroform to a final concentration of 100 *μ*g/mL. Furthermore, the C19:0 PL standard was dissolved in a 90 : 10 (v/v) mixture of chloroform and methanol, respectively, to maximize solubility. All IS stock solutions were transferred to GC sample vials, sealed, and stored in darkness at −20°C until use.

To identify the fatty acid methyl esters (FAMEs) resulting from the derivatization of FAs from* T. viridissima *and* Chorthippus *sp., a FAME mix containing 37 different FAMEs was chosen. The Food Industry FAME Mix was supplied by Restek (Bellefonte, PA, USA) and had a total concentration of 30 mg/mL. For further identification, the individual standards 14-methyl-pentadecanoate, 14-methyl-hexadecanoate, 16-methyl-heptadecanoate,* cis*-11-octadecenoic acid methyl ester, and hexacosanoic acid methyl ester were all purchased from Larodan AB, and* cis*-10-nonadecenoic acid methyl ester was supplied by Nu-Chek Prep, Inc. (MN, USA).

### 2.2. Samples and Sample Preparation

Wild individuals belonging to the species* T. viridissima*,* C. biguttulus*, and* C. brunneus* were all collected from the Asker area, Norway, during the period of June until October 2016. Individuals were macroscopically identified shortly after collection. Males and females are both represented within each species. No cleaning or treatment was carried out on any of the individuals after collection and subsequent storage at −20°C.

To prepare the insects for lipid extraction and analyses, the specimens were homogenized using liquid nitrogen and cryo pulverization. The homogenized sample material was then subjected to freeze-drying for 72 hours to remove all traces of water. Subsequently, the homogenized and dried samples were kept in the dark at −20°C.

### 2.3. Lipid Extraction and Methylation

For* T. viridissima*, the following amounts of sample material were weighed out for three consecutive sample preparations: 503 mg, 509 mg, and 508 mg, respectively. For* Chorthippus*, 252 mg and 251 mg were weighed out for two simultaneous sample preparations. Lipids were extracted according to a modified Folch et al. [26] procedure. In brief, 30 mL of a 2 : 1 chloroform and methanol (v/v) was transferred to a 100 mL Pyrex reagent bottle with a screw cap, along with 3.7 mL of C19:0 TG IS and 600 *μ*L of C11:0 TG IS solutions, the latter two by utilizing Hamilton syringes. The homogenized* T. viridissima* sample was added to the bottle, with subsequent shaking on an orbital shaker (Biosan PSU-10i, Riga, Latvia) at 450 rpm for 60 minutes in a horizontal position. The contents of the Pyrex bottle were then transferred to a separatory funnel through a porcelain Büchner filter funnel to retain larger pieces of sample material, and 6 mL 0.1 M NaCl in Milli-Q water was added. 10 mL of Folch's extraction mixture was used to wash the Pyrex bottle. The lower phase was decanted off into a glass beaker after shaking the separatory funnel vigorously until satisfactory separation of the two phases. Two additional liquid-liquid extractions were carried out with 4 mL chloroform and collected in the same glass beaker.

The organic phase was distributed equally to nine 6 mL Duran® GL 14 culture tubes. Removal of the solvent was carried out by inserting the glass tubes in heating blocks at 40°C in a pure nitrogen atmosphere until a dry residue remained. 1 mL of heptane was then added to each tube.

A sodium methoxide solution was prepared by dissolving metallic sodium, supplied by Merck (Darmstadt, Germany), in methanol to a final concentration of 5 mg/mL. 1 mL of the sodium methoxide solution was added to each of the glass tubes, with subsequent horizontal shaking for 20 minutes at 450 rpm. 1 mL of 10% boron-trifluoride-methanol solution was then added to each glass tube with an additional 20 minutes of shaking at 450 rpm. The glass tubes were subsequently heated in a water bath at 80°C for 20 minutes. The tubes were cooled to room temperature before the upper heptane phase of each tube was removed and collected in a single glass vial, which in turn was thoroughly mixed before transfer to three GC vials, ready for analysis by GC-MS.

*Chorthippus *sp. sample preparation was identical to the aforementioned method, with one minor modification: 20 mL instead of 30 Folch's extraction mixture was used for the initial lipid extraction, due to the lower amount of sample material used.

### 2.4. Solid Phase Extraction and Methylation

10 mL of Folch's extraction mixture was added to a 100 mL Pyrex reagent bottle with a cap, along with five internal standards: 600 *μ*L of C19:0 TG, 300 *μ*L of C11:0 TG, 200 *μ*L of C19:0 FFA, 100 *μ*L of C11:0 FFA, and 100 *μ*L of C19:0 PL, all transferred from their stock solutions using Hamilton syringes. Lastly, 100 mg of* T. viridissima* was added to the Pyrex bottle, before being subjected to horizontal shaking at 450 rpm for 60 minutes. The contents were poured through a funnel into a 100 mL separatory funnel, along with 3 mL of 0.1 M NaCl in Milli-Q water. The Pyrex bottle was washed with 5 mL of Folch's extraction mixture. The bottom phase in the separatory funnel was decanted into a glass beaker after rigorous shaking until separation of the two phases, followed by two additional liquid-liquid extractions using 2 mL chloroform. The contents of the glass beaker were distributed equally to twelve 6 mL Duran GL 14 culture tubes, along with the chloroform used to wash the beaker. The solvent was removed by inserting the twelve glass tubes in heating blocks at 40°C in a pure nitrogen atmosphere until dry residues remained, which were then dissolved by adding 1 mL of chloroform per tube. A vortex mixer was used to thoroughly mix the contents of each tube before transfer to twelve GC sample vials.

Two blank samples were also made for offline SPE by spiking 5 mL of chloroform with 100 *μ*L of C11:0 FFA IS and 200 *μ*L of C19:0 FFA IS and transferring 1 mL each to two GC sample vials. Six of the twelve sample vials were subjected to offline SPE along with the two blanks.

The offline SPE was carried out using a GX-274 ASPEC™ (Gilson, Middleton, WI, USA) and the accompanying software TRILUTION® LH Software version 3.0 (Gilson, Middleton, WI, USA). The method used for lipid fractionation was based on the previous work by Pinkart et al. [27] and Ruiz et al. [28]. Bond Elut NH_2_ 500 mg and 3 mL columns (Agilent Technologies, USA) were used as stationary phases and conditioned using 7.5 mL heptane. The samples (500 *μ*L) were applied to the columns, and NLs were eluted into glass vials using 5 mL chloroform, FFAs were eluted using 5 mL 98 : 2 diethyl ether : acetic acid (v/v), and PLs were eluted using 5 mL of methanol. The contents of the glass vials were then transferred to 6 mL Duran GL 14 culture tubes. FFAs eluted from the blank samples were also collected and transferred to glass tubes.

Preliminary testing of the offline SPE method revealed a contribution of the FFAs C14:0, C16:0, and C18:0 from the columns, which had to be accounted for by analysing blank samples and subsequently subtracting their mean areas from their respective counterparts in the FFA samples. However, no such contribution was detected for the NL and PL fractions.

The solvents in all tubes containing the three fractions, and blanks, were then evaporated to dryness in a nitrogen atmosphere at 40°C in heating blocks. 500 *μ*L of heptane was then added to each tube to dissolve the dry residues. The transesterification procedure was identical to the one previously described in Section 2.3. However, only sodium methoxide needed to be added to the fraction containing NLs, followed by 20 minutes of shaking at room temperature, in order to achieve methylation. In contrast to the NLs, a solution of 10% boron-trifluoride-methanol was added to the fraction containing FFAs and the blank samples, followed by shaking for 20 minutes, and heated in a water bath at 80°C for 5 minutes. The PL fraction followed the procedure of the latter but was heated for 20 minutes. The upper heptane phases of all fractions were then collected, homogenized on a vortex mixer, and redistributed to GC sample vials for analyses. The SPE fractionation was only performed for* T. viridissima* due to insufficient amount of sample material for* Chorthippus *sp.

### 2.5. GC-MS Analysis of FAMEs

The GC-MS method used was based on a previously published method [29]. An Autospec Ultima GC-MS (Micromass Ltd., Manchester, England) was used to identify the FAMEs in the samples. The MS was a three-sector instrument with EBE geometry. Electron ionization (EI) was used as the ionization method, accelerating the electrons to 70 eV before impact with the analyte molecules, and 40–600* m/z* was chosen as the mass range. Additionally, the mass spectrometer was tuned to a resolution of 1000. The temperature of both the ion source and transfer line was kept at 250°C. Full-scan acquisition mode was utilized.

Furthermore, the gas chromatograph used in combination with the MS was an Agilent HP6890 (Agilent Technology, Wilmington, DE, USA). Separation was carried out on a 60 m Restek column (Rtx®-2330) with 0.25 mm ID and a 0.2 *μ*m film thickness of fused silica biscyanopropyl cyanopropylphenyl polysiloxane stationary phase (Restek Corporation, Bellefonte, PA, USA). To inject the sample, a CTC PAL Auto Sampler was used (CTC Analytics AG, Zwinger, Switzerland), injecting 1.0 *μ*L at a split ratio of 1 : 10 into an injection chamber set to 250°C and using helium as a carrier gas (99,9999%, Yara, Rjukan, Norway) at a constant flow of 1 mL/min. The total run time was 92 minutes, with the initial GC oven temperature set to 65°C for 3 minutes, before increasing, at a rate of 40°C/min, to 150°C, and held for 13 minutes. Then it was held at 151°C for 20 minutes after increasing the temperature by 2°C/min. The temperature was then increased to 230°C, at a rate of 2°C/min and held for a total of 10 minutes. Finally, at a rate of 50°C/min, the temperature was held at 240°C for 3.7 minutes.

Undiluted triplicates were subjected to analysis by GC-MS after each sample preparation for the identification and quantitation of the complete FA profiles, with a single injection of each replicate. Two injections of heptane were carried out in-between each injection of a sample replicate. For the samples prepared using offline SPE, undiluted triplicates were made for each of the following fractions: NLs, PLs, and FFAs. Duplicates were made for the FFA blank samples. A single injection was carried out for each sample replicate, and two injections of heptane in-between each sample replicate.

The software used for the GC-MS analysis was Masslynx 4.0 (Waters, Milford, MA, USA), and NIST 08 Mass Spectral Library (Gaithersburg, MD, USA) was used to aid in the identification of FAMEs, along with the retention times of the standards present in Restek's Food Industry FAME Mix. Relative response factors previously determined by Devle et al. [29] were employed for quantitative determination.

## 3. Results and Discussion

### 3.1. FA Profile of* T. viridissima*

Only minor efforts have been made to explore the food potential of insect species from the Tettigoniidae family [20]. In this study a total of 37 different FAs were identified for* T. viridissima*, with C17:1*n*-7c being the only FA not to be detected in all replicates. The average concentrations of the FAs were converted to *μ*g/g of dry weight and are displayed in Table 1. Among the identified FAs, 29 of the 37 FAs were represented by reference standards and could thus be identified through both retention times and MS library searches. The remaining 8 FAs relied solely on MS library searches, based on returned values of match factor, reverse match factor, and probability, but they were identified in all replicates. The alkyl chain length varied from 12 to 26 carbon atoms, and SFAs, MUFAs, and PUFAs were all represented, including* n*-3 and* n*-6 FAs.

Several branched fatty acids, BCFAs, were also detected in low concentrations. Both* iso*- and* anteiso*-methyl branched FAs were present: 14-methylpentadecanoic acid, 14-methyl-hexadecanoic acid, 16-methylheptadecanoic acid, and 17-methyloctadecanoic acid.

The average total FA content for the bush cricket* T. viridissima* was found to be 10.4 g/100 g  ± 0.3 of sample dry weight. Previous studies show a large variation (8–48%) in lipid content for grasshoppers and crickets from this family [20]. Paul et al. [21] reported a fat content of 13% for the European bush cricket* C. discolor*. SFAs constituted 31.1% of the total FA content in* T. viridissima* and displayed the largest variation in data among the FA classes. MUFAs made up 35.9% and PUFAs 33.0% of the total FA content. Furthermore,* n*-3 FAs and* n*-6 FAs constituted 5.73% and 27.2%, respectively. The values for MUFAs and PUFAs also aligned with the values reported by Yang et al. [22]. In their study,* A. confirmata* (Gryllidae family) contained 33.5% MUFAs and 33.8% PUFAs. Although it is a species from a different family, a comparison is advantageous in confirming the plausibility of the data gathered in this study. However, the study by Paul et al. [21] reported approximately 19% SFAs, 21% MUFA, and 56% PUFAs for a different species in the Tettigoniidae family* (C. discolor)*. The differences in FA composition depend on a multitude of factors, for example, diet, species, and sex [20].

Most notably, C18:0, C16:0, C14:0, and their monounsaturated and polyunsaturated counterparts, including C18:1*n*-9c, C18:2*n*-6c, and C18:3*n*-3c, were by far the most abundant FAs in* T. viridissima*. Additionally, several C20 long-chain FAs were detected. The same trend was observed in the studies of lipids in the cricket* A. domesticus* by Hutchins and Martin [30] and Grapes et al. [23]. This trend was also found by Yang et al. [22] in* A. confirmata*. The fatty acids C18:0, C16:0, and C14:0 accounted for 3.44%, 25.4%, and 1.49%, respectively, of the total FA content in* T. viridissima*.

C18:1*n*-9 was the most abundant MUFA and FA, yielding 32.8% of the FA total. The two EFAs C18:2*n*-6c, LA, and C18:3*n*-3c, ALA, each contributed 26.6% and 5.60% of the total. Paul et al. [21] reported LA as being among the major FA constituents in the crickets* C. discolor* and* A. domesticus*. The FAs EPA, C20:5*n*-3c, and DHA, C22:6*n*-3c, have been extensively linked to several important functions in the human body, including prevention of cardiovascular diseases and inflammations [9]. While DHA was undetected in the samples, small amounts of EPA were identified and quantitated, accounting for 0.12% of the FA total. The precursor to both EPA and DHA, arachidonic acid (C20:4*n*-6c), accounted for less than 1%.

*n*-6/*n*-3-ratio of 4/1 has previously been linked to a 70% decrease in overall mortality [18]. However, this claim remains a subject of debate [31]. The calculated* n*-6/*n*-3-ratio for* T. viridissima* was 4.7/1, which is above the aforementioned value. According to Simopoulos [18], even an overall 5/1 ratio in the diet may provide beneficial effects for those affected by asthma. Additionally, a 5/1 ratio, or lower, has also been linked to decreased levels of serum cholesterol and proinflammatory cytokines [16]. The significant abundance of the EFA C18:2*n*-6c, as well as the high contents of MUFAs and PUFAs, would suggest that* T. viridissima* displays a nutritionally beneficial FA composition that could potentially impact positively on human health if incorporated into an already balanced diet. C18:1*n*-9c, OA, was overall the most abundant FA and is reported to have beneficial effects in patients suffering from diabetes II, as well as an ability to reverse the effects of inflammatory cytokines [32]. The presence of the other EFA, C18:3*n*-3c, further substantiates the claim that the FA composition of* T. viridissima* may positively affect human health.

Fractionation of the lipids in* T. viridissima* into NLs, FFAs, and PLs by use of offline SPE resulted in an average total FA content of 10.8 g/100 g  ± 0.3 of sample dry weight. This value is consistent with the total FA content reported for* T. viridissima* above. However, fewer FAs were found in the collated fractionated lipid profiles compared to the profile without fractionation. This is likely due to the introduction of an extra sample preparation step. All missing FAs were below 33 *μ*g/g d.w. in the unfractionated FA profile.

The total concentrations of the lipid classes are displayed in Table 2. NLs, including the storage lipids triacylglycerides, were by far the most abundant in* T. viridissima*, yielding a total concentration of 65.87 ± 2.67 mg/g of sample dry weight. PLs consistently yielded the lowest concentrations, with the exception of C18:0, and accounted for a total of 11.21 ± 0.48 mg/g d.w. The overall lower content of PLs is attributed to the primary role of phospholipids as constituents of the cell membrane. The FFAs, however, constituted a total of 31.47 ± 1.19 mg/g d.w., a far higher concentration than previously anticipated. Furthermore, the FAs C16:0 and C18:0 constituted the majority of the total SFA content within each respective fraction. The MUFA C18:1*n*-9c and the PUFAs C18:2*n*-6c and C18:3*n*-3c were all present in major quantities within each fraction. The precision was deemed satisfactory, thus demonstrating that SPE could become a useful method in future lipid studies of other insects.

### 3.2. FA Profile of the Combined* Chorthippus* Species* C. brunneus* and* C. biguttulus*

A total of 33 FAs were identified for* C. brunneus *and* C. biguttulus*. Of these, 25 FAs were identified using the retention times of reference standards, as well as searches in the MS library. The remaining 8 relied upon the MS library alone for identification, but they were present in all replicates. The results for each FA were converted to *μ*g/g dry weight and are presented in Table 1. As with the FAs present in* T. viridissima*, the chain length varied from 10 to 26 carbon atoms, and SFAs, MUFAs, and PUFAs were all identified among the FAs, including* n*-6 and* n*-3 FAs. In contrast to* T. viridissima*, no BCFAs were detected. The SFAs C10:0, C21:0, and C22:0 were not detected in the* T. viridissima* replicates but were present in* Chorthippus *sp. in average concentrations of 13.5, 15.1, and 26.2 *μ*g/g d.w., respectively.

**Figure 1:**
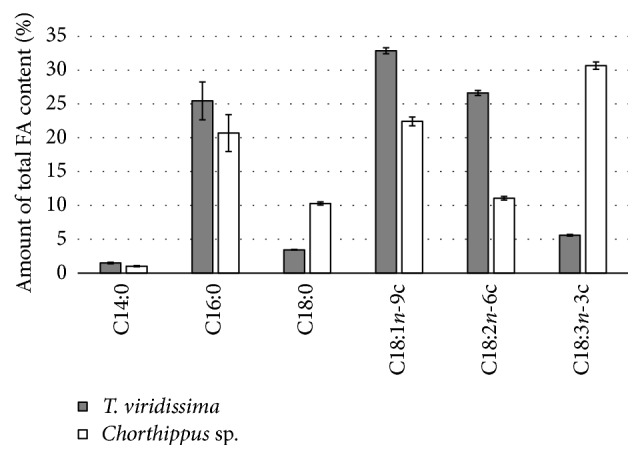
A graphical representation of the average percentages of each of the most abundant FAs found in both* T. viridissima* and* Chorthippus *sp., relative to total concentration of FAs per gram of sample dry weight. For* T. viridissima*, *n* = 9; for* Chorthippus*, *n* = 6.

The average total FA content of* Chorthippus *sp. was 6.14 g/100 g  ± 0.17 of sample dry weight, a value lower than what was found for* T. viridissima*. Paul et al. [21] reported a total FA content of 10% of dry matter for the species* C. parallelus*, which belongs to the same genus. However, a variation in lipid content from 4 to 22% for species in the Acrididae family has been reported [20], and interspecies differences for total FA contents are to be expected. There were significant, quantitative differences in the FA contents of* Chorthippus *sp. and* T. viridissima*, displayed in Table 3. SFAs in* Chorthippus *sp. constituted 32.7% of the total amount of FAs present, a similar value to the SFA content of* T. viridissima*. The MUFA content in* Chorthippus *sp. was lower, accounting for 25.1% of the FAs, and PUFAs constituted a total of 42.1%. The same values for* T. viridissima* were 35.9 and 33.0%, respectively. These differences are largely explained by the variations of the following three FAs: C18:1*n*-9c, C18:2*n*-6c, and C18:3*n*-3c, as shown in Figure 1.

These three FAs accounted for the majority of the total FA content. While* Chorthippus *sp. contained comparatively lower amounts of C18:1*n*-9c and C18:2*n*-6c, the FA C18:3*n*-3c accounted for 30.6% of the total FA content. C18:1*n*-9c and C18:2*n*-6c, however, respectively, contributed 22.4 and 11.1%. The higher concentration of C18:3*n*-3c, ALA, in* Chorthippus *sp. is likely due to the herbivorous diet, as opposed to the more carnivorous diet of* T. viridissima. *The results of Paul et al. [21] also proved C18:3*n*-3c to be present in major quantities in* C. parallelus*, and the authors similarly concluded that the diet was responsible for the abundance of ALA in* C. parallelus*.

The SFAs C14:0, C16:0, and C18:0 were the major contributors to the total SFA content of* Chorthippus *sp., each accounting for 10.3, 20.7, and 1.01% of the total FA amount. The same trend was observed in the case of* T. viridissima*. A graphical representation of these FAs, in both* T. viridissima* and* Chorthippus *sp., is displayed in Figure 1.

The* n*-6/*n*-3 ratio of* Chorthippus *sp. was 0.36/1, which corresponds to what was reported by Paul et al. [21] for* C. parallelus* (0.33). This is a more favourable ratio from a nutritional point of view than the ratio calculated for* T. viridissima* and below the ratio of 1 suggested by Simopoulos [18]. The concentration of the essential* n*-3 FA C18:3*n*-3c was also 5.4 times higher in* Chorthippus *sp. The other EFA, C18:2*n*-6c, was, however, 2.4 times higher in* T. viridissima*. The nutritionally beneficial FA composition of* T. viridissima* emphasized the high contents of the EFA C18:2*n*-6c, as well as the beneficial effects of its most abundant FA: C18:1*n*-9c. However, the higher PUFA content, the significantly higher quantities of the EFA C18:3*n*-3c, and the more favourable* n*-6/*n*-3 ratio would suggest that* Chorthippus *sp. exhibit the more health beneficial FA composition, comparatively, as a potential human food ingredient.

## 4. Conclusions

The results presented in this study highlighted the quantitative diversity of FAs for different species belonging to the order Orthoptera. Significant differences in the contents of MUFAs and PUFAs in the bush cricket* T. viridissima* and grasshoppers* Chorthippus *sp. were observed, as well as differences in the total FA contents. The FA contents were 10.4 and 6.14 g/100 g of dry matter, respectively.* Chorthippus *sp. were richer in PUFAs (42.1%) than* T. viridissima* (33.0%) and contained higher amounts of the EFA C18:3*n*-3c (30.6%). In contrast,* T. viridissima* was richer in the EFA C18:2*n*-6c (26.6%) and C18:1*n*-9c (32.9%). Fractionation of the lipids in* T. viridissima* into neutral lipids, free fatty acids, and polar lipids resulted in a total FA content of 10.8 g/100 g. The average concentrations of the three fractions were 65.87, 31.47, and 11.21 mg/g of dry matter, respectively. The abundance of FAs potentially beneficial to human health, high contents of MUFAs and PUFAs relative to SFAs, and favourable* n*-6/*n*-3 ratios suggest that both the* Chorthippus* species and* T. viridissima* display favourable nutritional profiles, although further studies are needed to conclusively mark all three species as safe for human consumption.

## Figures and Tables

**Table 1:** The complete fatty acid profiles of both *T. viridissima* and *Chorthippus *sp., in order of elution. The average concentrations for *T. viridissima* are the result of three sample preparations, each containing three parallels. The average concentrations for *Chorthippus *sp. are the result of two sample preparations, each containing three parallels. All values are presented as *µ*g/g of sample dry weight.

FA	Average ± SD (*µ*g/g d.w.)	
	*T. viridissima*	*Chorthippus *sp.
C10:0	n.d.^b^	13.52 ± 0.56
C12:0	72.80 ± 4.62	113.3 ± 5.6
C13:0	10.81 ± 1.25	7.15 ± 1.63
C14:0	1547 ± 110	622.2 ± 54.2
C14:1*n*-3c^a^	6.23 ± 0.44	2.78 ± 0.48
C14:1, other^a^	3.94 ± 0.48	n.d.^b^
C14:1*n*-5c	15.94 ± 0.63	4.57 ± 0.48
C15:0	31.24 ± 1.69	19.28 ± 0.89
C15:0 (14-methyl)	55.23 ± 4.82	n.d.^b^
C16:0	26475 ± 2903	12723 ± 1673
C16:1*n*-9t^a^	7.17 ± 0.73	6.46 ± 0.36
C16:1 other^a^	68.54 ± 3.02	103.4 ± 5.6
C16:1*n*-7c	1854 ± 61	274.3 ± 16.9
C16:1*n*-5c^a^	n.d.^b^	28.23 ± 1.43
C16:0 (14-methyl)	59.86 ± 2.43	n.d.^b^
C17:0	109.2 ± 3.4	248.5 ± 14.3
C16:2*n*-6t^a^	8.88 ± 1.33	15.90 ± 2.42
C17:1*n*-7c	71.57 ± 1.98	66.19 ± 2.86
C17:0 (16-methyl)	149.5 ± 2.5	n.d.^b^
C18:0	3576 ± 52	6321 ± 143
C18:1*n*-9c	34178 ± 458	13776 ± 397
C18:1*n*-7c	505.2 ± 5.9	359.8 ± 14.3
C18:1*n*-8t^a^	n.d.^b^	20.08 ± 0.88
C18:0 (17-methyl)^a^	33.15 ± 2.80	n.d.^b^
C18:2*n*-6c	27693 ± 390	6798 ± 168
C19:1 other^a^	n.d.^b^	15.50 ± 0.89
C19:1*n*-9c	73.27 ± 5.96	26.63 ± 1.68
C18:3, other^a^	14.21 ± 2.00	55.06 ± 4.14
C20:0	172.9 ± 25.7	n.d.^b^
C18:3*n*-3c	5818 ± 135	18845 ± 339
C20:1*n*-11c^a^	26.70 ± 3.89	73.15 ± 1.77
C20:1*n*-9c	357.2 ± 11.8	654.1 ± 28.1
C21:0	n.d.^b^	15.09 ± 1.12
C20:2*n*-6c	124.9 ± 3.4	44.13 ± 2.02
C22:0	n.d.^b^	26.18 ± 0.82
C20:3, other^a^	10.85 ± 0.92	n.d.^b^
C20:3*n*-6c	19.07 ± 11.66	n.d.^b^
C20:3*n*-3c	n.d.^b^	124.8 ± 4.0
C20:4*n*-6c	519.7 ± 17.3	n.d.^b^
C22:1*n*-9c	141.1 ± 6.3	27.65 ± 1.60
C20:5*n*-3c	126.4 ± 34.9	n.d.^b^
C24:0	26.95 ± 3.21	6.06 ± 0.46
C24:1*n*-9c	13.14 ± 1.84	n.d.^b^
C26:0	13.60 ± 3.21	12.52 ± 0.56

^a^The FA is not confirmed by a reference standard.  ^b^n.d.: not detected.

**Table 2:** The average concentration and standard deviation, of each fatty acid across the three lipid fractions: neutral lipids, free fatty acids, and polar lipids. The results are based on three sample preparations, each including three parallels of each fraction, for *T. viridissima*. All values are presented as *µ*g/g of dry matter.

FA	Average ± SD [*µ*g/g d.w.]		
	NLs	FFAs	PLs
C12:0	87.96 ± 12.79	n.d.^b^	n.d.^b^
C14:0	1311 ± 122	324.9 ± 52.1	85.86 ± 23.25
C16:0	21466 ± 2576	8252 ± 765	3829 ± 325
C16:1 ^a^, other	52.66 ± 6.12	n.d.^b^	n.d.^b^
C16:1*n*-7c	1112 ± 50	334.1 ± 73.4	n.d.^b^
C17:0	76.86 ± 10.82	77.35 ± 14.08	n.d.^b^
C17:1*n*-7c	46.82 ± 10.00	46.40 ± 11.74	n.d.^b^
C17:0 (16-methyl)	96.88 ± 14.77	n.d.^b^	n.d.^b^
C18:0	1212 ± 59	1954 ± 214	2694 ± 145
C18:1*n*-9c	23018 ± 623	7613 ± 545	2535 ± 214
C18:1*n*-7c	248.3 ± 9.5	142.8 ± 25.2	n.d.^b^
C18:2*n*-6c	13652 ± 285	10953 ± 691	1927 ± 247
C19:1*n*-9c	44.34 ± 4.23	n.d.^b^	n.d.^b^
C20:0	80.96 ± 5.77	88.49 ± 76.13	n.d.^b^
C18:3*n*-3c	2961 ± 125	1352 ± 93	145.8 ± 15.9
C20:1*n*-9c	171.3 ± 12.2	53.34 ± 9.18	n.d.^b^
C20:2*n*-6c	46.99 ± 6.08	33.81 ± 7.89	n.d.^b^
C20:4*n*-6c	108.6 ± 9.7	168.9 ± 17.5	n.d.^b^
C22:1*n*-9c	74.36 ± 13.65	31.31 ± 8.13	n.d.^b^
C20:5*n*-3c	n.d.^b^	45.57 ± 5.23	n.d.^b^

*Total [mg/g]*	65.87 ± 2.67	31.47 ± 1.19	11.21 ± 0.48

^a^The FA is not confirmed by a reference standard.  ^b^n.d.: not detected.

**Table 3:** A comparison of the major fatty acid classes found in both *T. viridissima* and *Chorthippus *sp. All values are presented as mg/g of sample dry weight and as (%) of total fatty acid content.

FA class	Average ± SD [mg/g d.w.]	
	*T. viridissima*	*Chorthippus *sp.
Σ SFAs	32.33 ± 2.90 (31.1%)	20.13 ± 1.68 (32.7%)
Σ MUFAs	37.32 ± 0.46 (35.9%)	15.44 ± 0.40 (25.1%)
Σ PUFAs	34.33 ± 0.41 (33.0%)	25.88 ± 0.37 (42.1%)
Σ*n*-3 FAs	5.96 ± 0.14 (5.73%)	19.02 ± 0.34 (31.0%**)**
Σ*n*-6 FAs	28.36 ± 0.39 (27.2%)	6.86 ± 0.08 (11.1%)

SFAs = saturated fatty acids, MUFAs = monounsaturated fatty acids, and PUFAs = polyunsaturated fatty acids.
